# Four Cases of Cysts in the Neck of Similar Character, and Possibly Developed in Connection with Branchial Clefts

**Published:** 1911-06

**Authors:** Charles A. Morton

**Affiliations:** Professor of Surgery in the University of Bristol, Senior Surgeon to the General Hospital and the Children's Hospital, Consulting Surgeon to the Cossham Memorial Hospital.


### FOUR CASES OF CYSTS IN THE NECK OF SIMILAR CHARACTER, AND POSSIBLY DEVELOPED IN CONNECTION WITH BRANCHIAL CLEFTS.

BY

CHARLES A. MORTON, F.R.C.S.,

*Professor of Surgery in the University of Bristol,*
*Senior Surgeon to the General Hospital and the Children's Hospital,*
*Consulting Surgeon to the Cossham Memorial Hospital.*

---

DURING the last few years these cases have come under my care. All the patients presented soft, fluctuating swellings just below the angle of the jaw and under the sterno-mastoid. In one case the cyst was of several years' duration, in the others of several months, and in all they were quite painless. The question of diagnosis from chronic tuberculous abscess had to be carefully considered. The absence of any solid enlarged glands was against the diagnosis of gland abscess, but did not of course exclude it, and a certain diagnosis could not be made between the two conditions before operation. In my last case an operation had been performed some years before the patient came under my care for excision of tuberculous glands at another hospital, and a very small firm gland could be felt under the scar on that side of the neck. This was, of course, rather strong evidence that any chronic fluctuating swelling on the other side of the neck was also a gland abscess, and yet the swelling was so like the other three cysts I had removed that it seemed to me quite likely it might prove to be of that nature, as it did.

The possibility of a sub-fascial lipoma had also to be considered in the differential diagnosis of these cysts, and as I was dissecting out one of them it looked so like a lipoma I thought that it was of that nature. The yellow pea-soup-like contents showed very clearly through the very thin cyst wall which remained after the outer layers were peeled off, and caused the striking resemblance. After removal it was by no means easy to say what form of cyst they were. The contents were not the sebaceous material described in the books as present in branchial cysts (when they do not contain mucus). In one cyst it resembled pea-soup, and in another was so like pus that I thought when I saw it cut open in a basin, before I was myself able to examine its wall, that I must have removed a chronic tuberculous gland abscess, and in another case it was just like very pale liquid fæces. But the fluid which looked so like pus did not present its microscopic characters, as there were comparatively few cells, but many oil globules.

During the dissection out of the cyst, I was conscious of peeling off more than one layer of cyst wall, the outermost being rather firmly attached to the surrounding deep structures ; but eventually I came on a very thin-walled sac, almost the whole of which could be shelled out. This wall was so thin in places as to be transparent, and on its inner surface in some of the cysts, were raised white bodies, almost the size of small pin heads, and suggesting miliary tubercles. On the inner wall of other cysts these were confluent, forming raised white patches of some size. In three of the cysts the wall was examined microscopically, and showed a lining of layers of flattened epithelium. In one cyst there was quite a thick piece of tissue in the wall, and this resembled a portion of lymphatic gland under the microscope.

Cysts arising in unobliterated portions of the branchial clefts are not usually congenital, but are stated by some surgical writers to occur about puberty, by others before thirty years of age, but occasionally quite late in life. So that the age of the older patients would not be too great for branchial cysts.

There is no other recognised cyst of the neck with which these can be classified. They are entirely different from cystic hygroma, or, as they are sometimes termed, "lymphatic cysts." There is certainly no proof that these cysts arose through failure in obliteration of a portion of a branchial cleft; they have more the character of cysts believed to arise in that way than any other form of cysts of the neck, but they were not adherent to either the hyoid bone or the pharynx, as such cysts are stated often to be. It seems, however, well to carefully record these cases, for such cysts are very likely to be considered to be chronic gland abscesses; and if they are not branchial cysts, these records, when compared with the records of other cases of the same kind, may throw light on their nature. In all my four cases the cysts were on the left side.

**Case 1.**—L. S., female, aged 30. March, 1908. General Hospital. Soft, painless, fluctuating swelling, size of hen's egg, just below angle of jaw on left side, under anterior border of sterno-mastoid. Short duration There were no solid glands to be felt, and the diagnosis lay between gland abscesses of tuberculous nature and a cyst. At the operation a cyst was found lying on the large vessels. It contained pea-soup-like material. Several layers of flattened epithelium were found on the inner wall. Primary union.

**Case 2.**—C. P., female, aged 21. November, 1910. General Hospital. Swelling in neck began eight months before admission. There was a very soft, fluctuating swelling, size of a hen's egg, under the left angle of the jaw and anterior border of sterno-mastoid. No solid glands could be felt. The cyst lay on the deep vessels of the neck. This is the case in which it resembled a lipoma during removal. The character of the cyst wall has been already described. Primary union.

**Case 3.**—W. V., male, aged 18. February, 1911. General Hospital. He noticed the lump in his neck, the size of a marble, four months before admission. It had been painted with iodine before admission. There was an oval swelling, the size of a duck's egg, lying under the upper part of the left sterno-mastoid, bulging in front of its anterior border, but also extending into the posterior triangle. It was softly fluctuating, and quite smooth. No solid glands could be felt on that side of the neck, and there was only one, the size of a shot, in the posterior triangle on the other side. This was the case in which the cyst contained fluid resembling pus. The structure of the cyst has been already described. Primary union.

**Case 4.**—J. W., female, aged 30. Women's Ward of Children's Hospital. March, 1911. Six years before admission she had tuberculous glands removed from right side of neck at St. Thomas's Hospital. Information as to this was kindly furnished by the Registrar at St. Thomas's Hospital. At the same time she says she had a lump in the left side of the neck, and this had grown into its present size since, more especially in the last three years. She never had any pain from it. There was a very soft, fluctuating, smooth swelling just below the left angle of the jaw, and under the anterior border of the sterno-mastoid. There were no solid glands on that side of the neck, but there was a small one under the scar, where glands had been excised on the other side. This was the case in which the cyst contained fluid like pale liquid fæces. The character of the cyst has been already described. Primary union.

